# Magnetic coagulometry: towards a new nanotechnological tool for *ex vivo* monitoring coagulation in human whole blood†

**DOI:** 10.1039/d3nr02593d

**Published:** 2023-12-21

**Authors:** Antonio Santana-Otero, Alan Harper, Neil Telling, Daniel Ortega, David Cabrera

**Affiliations:** a Condensed Matter Physics Department, Faculty of Sciences, University of Cádiz Campus Universitario Rio San Pedro s/n 11510 Puerto Real Cádiz Spain daniel.ortega@uca.es; b School of Medicine, Keele University Newcastle-under-Lyme Staffordshire. ST5 5BG UK; c School of Pharmacy and Bioengineering, Keele University, Guy Hilton Research Centre Thronburrow Drive Hartshill Stoke on Trent ST47QB UK d.c.cabrera@keele.ac.uk; d iMdea Nanociencia Campus Universitario de Cantoblanco. C/Faraday 9 28049 Madrid Spain; e Institute of Research and Innovation in Biomedical Sciences of Cádiz (INiBICA), University of Cádiz 11002 Cádiz Spain

## Abstract

Blood clotting disorders consisting of unwanted blood clot formation or excessive bleeding are some of the main causes of death worldwide. However, there are significant limitations in the current methods used to clinically monitor the dynamics of clot formation in human whole blood *ex vivo*. Here a new magnetic coagulometry platform for testing *ex vivo* coagulation is described. This platform exploits the sensitivity of the out-of-phase component of alternating current (AC) magnetic susceptibility (*χ*′′) to variations in mobility and agglomeration of magnetic nanoparticles when trapped during blood clot formation. By labelling human whole blood with magnetic nanoparticles, the out-of-phase component of AC magnetic susceptibility shows that the dynamics of blood clot formation correlates with a decrease in the out-of-phase component *χ*′′ over time activation of coagulation. This is caused by a rapid immobilisation of nanoparticles upon blood coagulation and compaction. In contrast, this rapid fall in the out-of-phase component *χ*′′ is significantly slowed down when blood is pre-treated with three different anticoagulant drugs. Remarkably, the system showed sensitivity towards the effect of clinically used direct oral anticoagulation (DOAC) drugs in whole blood coagulation, in contrast to the inability of clinical routine tests prothrombin time (PT) and partial thromboplastin time (PTT) to efficiently monitor this effect. Translation of this nanomagnetic approach into clinic can provide a superior method for monitoring blood coagulation and improve the efficiency of the current diagnostic techniques.

## Abstract 2

Blood clotting disorders are among the main causes of death worldwide. Magnetic coagulometry provides a new method to test coagulation in human whole blood *ex vivo*, offering grounds for the improvement of current methods in research and clinic.

## Graphical abstract

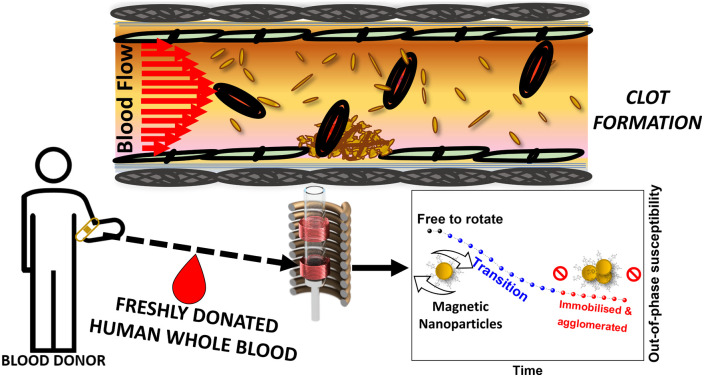

## Introduction

**Fig. 1:**
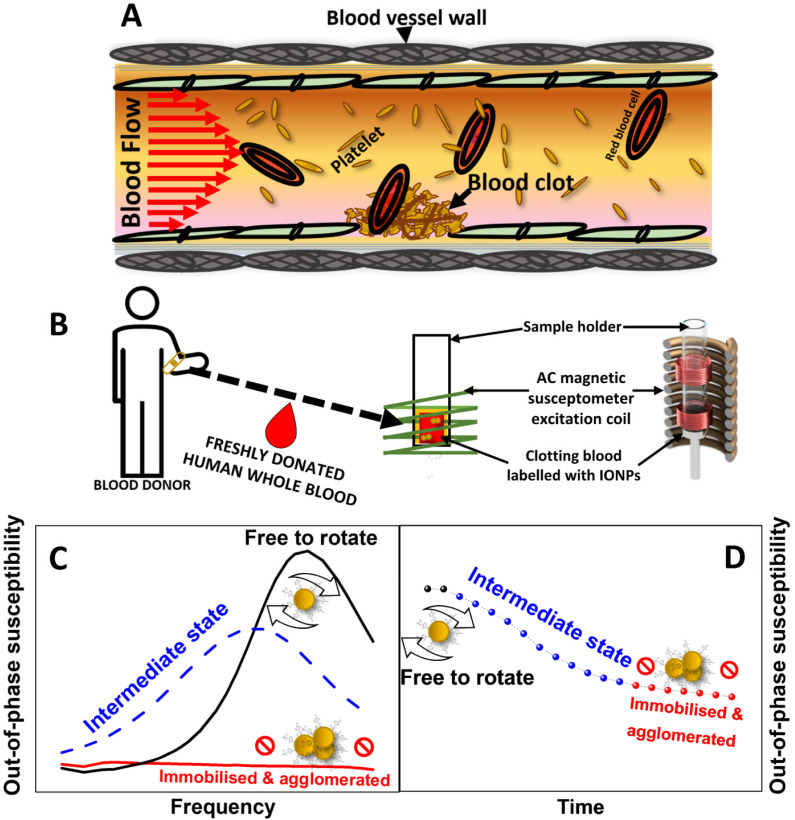
Principles of AC magnetic susceptibility provide a new method for monitoring *ex vivo* blood coagulation in human whole blood. (A) Schematic representation of haemostasis in a blood vessel. (B) Schematic representation of AC magnetic susceptibility measurements of IONPs-labelled whole blood. (C) Typical behaviour of the out-of-phase component of AC susceptibility in measurement performed in a frequency spectrum or (D) at fixed frequency over time, upon agglomeration and immobilisation of magnetic nanoparticles in clotting whole blood.

Bleeding and thrombotic disorders such as haemophilia, stroke and venous thromboembolism (VTE) are among the main causes of death and disability globally.^1,2^ Normally blood clotting occurs through a physiological process called haemostasis (Fig. 1A). Upon vascular injury, a blood clot is formed at the site of damage to plug rapidly and prevent further blood loss. A blood clot is formed through both the platelet activation at the site of injury,^3^ as well the localised formation of fibrin through controlled activation of a series of linked enzymatic reactions called the coagulation cascade.^4^ After the damaged wall is patched and repaired, the targeted activation of plasmin on fibrin and surrounding endothelial cells triggers the regulated resolution of the clots through destruction of fibrin (a process called fibrinolysis).^5^ However, bleeding disorders can arise when platelets become dysfunctional, or production of clotting factors prevent the normal functioning of the coagulation cascade. Conversely, thrombotic disorders occur when these components of the haemostatic system become inappropriately activated, such as upon rupture of atherosclerotic plaques.

Treatment of haemostatic disorders are generally addressed through the administering of pro- or anti-thrombotic drugs^6^ or blood products.^7^ However, the effectiveness of these formulations strongly depends on reliable methods for assessing blood coagulation. Clinically, routine *ex vivo* tests of blood clotting mainly consists of prothrombin (PT) and partial thromboplastin time (PTT). However, these only test the activity of the coagulation cascade in cell-free plasma samples. As these tests lack the presence of platelets and other blood cells, they solely report on the coagulability on plasma, without providing any information on clot formation, compaction, and susceptibility to clot-busting drugs. This has led to the effectiveness of these tests in detecting the efficacy of direct oral anticoagulants (DOAC) in coagulation being questioned.^8^ These tests are of limited use in complex patients such as those suffering from trauma,^9^ and are not reliable for the diagnosis of many haemostatic disorders.^10,11^

Currently thromboelastography (TEG) and rotatory thromboelastography (ROTEM) are used for global assessments of whole blood coagulation, which are dependent upon the activation of platelets, the coagulation cascade, and the fibrinolytic system. Both systems place a detection pin in a cup of coagulating blood. To assess changes in the viscoelasticity of the blood sample, the cup is rotated in TEG, whilst the detecting pin is rotated in ROTEM. As the blood clots, it adheres to the pin while the viscoelasticity of the blood increases, causing increased deflection of the pin relative to the cup and allowing clotting to be measured. Despite initial interest in their potential clinical applications, there are significant limitations to both TEG and ROTEM that reduces their capacity to detect and diagnose haemostatic conditions.^12^ In this regard, TEG and ROTEM only detect coagulation at a macroscopic level by testing mechanical properties of the clot as a single entity. However, the internal structure and mechanical properties of clots evolve during coagulation, and these changes can strongly impact on the effectiveness of pro- or anti-thrombotic treatments.^13^ Additionally, the large operational costs associated with these techniques impedes their wider usage^14^ and restricts it to critical care and emergency surgery.^15^

A method that allows assessment of coagulation of whole human blood by potentially probing mechanical changes in the clot in the nanoscale in real time, would significantly boost our understanding of the dynamics of coagulation, and how this determines the effectiveness of pro- and anti-thrombotic drugs.

AC magnetic susceptibility (ACS) is a physical property characteristic of magnetic nanoparticles ruled by two physical relaxation processes: Néel and Brownian relaxation. While the Néel relaxation concerns the spontaneous reorientation of the magnetic moment inside the nanoparticles,^16^ Brownian relaxation involves the spatial reorientation of the nanoparticles by rotational diffusion when these are suspended in liquids.^17^ The latter mechanical rotation is modulated by both the size of the aggregates that nanoparticles form in a liquid medium, and the viscosity of the liquid the nanoparticles are dispersed in. Therefore, a characteristic Brownian relaxation time can be estimated using the equation *τ*_B_ = 3*ηV*_H_/*k*_B_*T*, where *η* is the viscosity of the dispersion medium, *V*_H_ is the hydrodynamic volume of the nanoparticles in the liquid media, *k*_B_ is the Boltzmann constant and *T* the temperature.

Relaxation processes in magnetic nanoparticles can be tracked using AC magnetic susceptibility measurements. These measurements are usually described in terms of complex AC susceptibility *χ*_AC_ = *χ*′ + *χ*′′, where *χ*′ is the in-phase (or real) component and *χ*′′ is the out of phase (or imaginary) component of the magnetic susceptibility. Interestingly, changes to the Brownian relaxation time in nanoparticles can be detected using this technique by recording the out-of-phase component of susceptibility (*χ*′′). By following the displacement and widening of a peak related to Brownian relaxation to lower frequencies until its eventual fading (Fig. 1C), *χ*′′ measurements recorded in a spectrum of magnetic field frequencies reveal the immobilisation of magnetic nanoparticles. Consequently, the immobilisation of magnetic nanoparticles can be also evidenced by measurements of *χ*′′ at a fixed frequency by recording variations of this component over time (Fig. 1D). Following this rationale, we have previously demonstrated the suitability of this technique to probe the immobilisation of magnetic nanoparticles either in viscous media, when the nanoparticles are internalised in cell cultures,^18–20^ or when interacting with the surface of a blood clot.^21^

Building on this principle and by labelling whole human blood samples with magnetic nanoparticles, it should be possible to create a new method based on ACS for monitoring the viscosity and aggregation of nanoparticles into the growing clot (Fig. 1B). Here we propose a magnetic coagulometry technique that combines the use of AC magnetic susceptometry and sucrose-coated iron oxide nanoparticle (IONPs) as nanoprobes to test coagulation in freshly donated whole human blood (Fig. 1B). Our work intends to provide a proof of concept for the application of nanomagnetism for real-time monitoring of blood coagulation, holding sufficient promise of a more effective method for clinical assessment of blood clotting disorders.

## Experimental

### Nanoparticle synthesis

Nanoparticles synthesis was carried out in a Schlenk line following a modified version of the co-precipitation method of iron salts described by Massart *et al.*^22^ 0.8 mmol of iron(ii) chloride hexahydrate, 1.6 mmol of iron(iii) chloride anhydrous and 0.8 mmol of sucrose were dissolved in 20 mL of distilled water in a 100 mL rounded flask. The temperature was then gradually raised to 60 °C under continuous magnetic stirring at 600 rpm, after which dissolved oxygen from the solution was purged by generating vacuum thrice. Immediately after, the temperature of reaction was progressively raised under a protective N_2_ atmosphere up to 100 °C, injected then with 135 μL of a saturated solution of Na_2_CO_3_ drop-wise with a syringe, and left 1 hour under these conditions. After that, the reaction was cooled down to room temperature and washed five times with Double Distilled Water (ddH_2_O) *via* magnetic separation with a NdFeB magnet.

### Structural, morphological and magnetic characterisation of IONPs

#### X-ray diffraction (XRD)

Diffractograms were carried out with a Rigaku Smart lab diffractometer, using Cu K-alpha1 [Å] = 1.54060, K-alpha2 [Å] = 1.54443, and K-beta [Å] = 1.39225. The samples were magnetically separated and dried with a N_2_ flux in an XRD glass sample holder. Scattering from samples was measured from 5° to 100° with a step size of 0.0264 2q and scan time of 1 s.

#### Transmission electron microscopy (TEM)

Copper TEM grids coated with ultrathin 3–4 nm carbon films were immersed in nanoparticle water suspensions for a few seconds, retrieved and left to fully dry. TEM were performed in a JEOL JEM-1010 microscope operated at 100 kV and equipped with a CMOS 4 K × 4 K, F416 de TVIPS camera. The median core size of nanoparticles was determined from the resulting statistical size distribution upon measuring the projected diameter using Image J.^23^

#### Dynamic light scattering (DLS)

Hydrodynamic size *D*_H_ of magnetic nanoparticles suspended in ddH_2_O and in 2.5, 5 and 10% of water/glycerol solutions (v/v) was measured with a Zetasizer Nano ZS (Malvern Panalytical, UK). To prevent any disparity in *D*_H_ due to IONPs concentration with the rest of the experiments, the nanoparticle colloids were placed in small volume, disposable plastic cuvettes at a similar Fe concentration to that used in AC magnetic susceptibility experiments (0.066 mg_Fe_ mL^−1^).

#### Nano tracking analysis

For determining the number of IONPs per volume in the colloids, measurements were performed in a Nanosight NS300 (Malvern-Panalytical, UK). Samples with an initial concentration of 2.2 g L^−1^ were diluted to 2 : 5000 and injected into the instrument chamber using a 1 mL syringe. The video data was collected for 60 seconds and repeated six times.

#### Inductively coupled plasma optical emission spectrometry (ICP-OES)

Iron concentration within a concentrated stock of IONPs water colloidal solution was determined using inductively coupled plasma optical emission spectrometry in the Instituto de Ciencia de Materiales-CSIC, Madrid (Spain). 50 μL aliquots from the IONPs suspensions were mixed with 2 mL of HNO_3_ in a 50 mL volumetric flask until complete digestion was reached at room temperature. Afterwards, samples were diluted 1 : 1000 in water and measured in a PerkinElmer spectrometer, model OPTIME 2100DV, using wavelengths of 238.204 and 239.562 nm. Iron concentration was determined by comparing with a standard iron solution.

### Blood preparation

This study was conducted according to the guidelines of the Declaration of Helsinki and approved by Keele University Research Ethics Committee (project reference MH-200154, ERP2335). The samples were acquired, stored, used and disposed in accordance with the Human Tissue Act under the Keele University Human Tissue License. Blood was collected by venepuncture from healthy volunteers who had given written informed consent. Blood was mixed in a proportion of 9 parts blood : 1 part 3.8% [w/v] sodium citrate solution and used in experiments the same day of collection.

### AC magnetic susceptibility

An in house-built AC magnetic susceptometer was used to characterise the magnetic behaviour of nanoparticles. This prototype has been thoroughly validated through previous studies in our labs.^18–21,24^ Two different measurement modes were used to perform AC susceptometry measurements: (i) a frequency spectral mode entailing measurements of the in- and out-of-phase components of AC magnetic susceptibility from 1 Hz up to 10 kHz, and (ii) a single frequency mode for measuring the out-of-phase component from AC magnetic susceptibility at 400 Hz every 1.5 minutes, over a period of 23 minutes. Building on our previous studies with this technique,^18–21,24^ and aiming at minimising the nanoparticle dose while still obtaining a reliable ACS signal, a concentration of 0.066 mg_Fe_ mL^−1^ IONPs was used in the experiments.

#### Frequency spectral mode

0.066 mg_Fe_ mL^−1^ of IONPs was added into 200 μL of (i) ddH_2_O, (ii) ddH_2_O and glycerol 2.5, 5, 10, 50 and 97.5% (v/v) solutions, or (iii) non-recalcified (*i.e.* non-actively clotting) freshly-donated human blood and measured in frequency spectral mode. For determining the AC magnetic susceptibility response of IONPs in actively clotting blood, repeated measurements of IONPs suspended in clotting blood were performed. 200 μL blood samples containing 0.066 mg_Fe_ mL^−1^ IONPs were re-calcified with 20 mM of CaCl_2_, stimulated with 125 μM adenosine diphosphate (ADP) for triggering coagulation and placed in the sample holder inside the instrument. Measurements were then triggered and performed continuously in intervals of 45 minutes for 3 hours and 45 minutes, and after 24 h. All the measurements were preceded by incubation of IONPs in each aqueous solution or blood at 37 °C for 5 minutes.

#### Single frequency mode

200 μL whole blood was pre-treated with different doses of the anticoagulant drugs heparin, rivaroxaban or bivalirudin; or the anti-platelet drug MRS 2179. 200 μL of freshly donated human whole blood labelled with 0.066 mg_Fe_ mL^−1^ IONPs was incubated with each of these drugs at different concentrations at 37 °C for 5 minutes. After that, blood clotting was triggered in a similar fashion to that used for frequency spectral mode experiments. Control samples were treated with the respective drug carriers in each experiment. In experiments where the intrinsic pathway was inhibited (Fig. 9), whole blood samples were pre-treated with 50 μg mL^−1^ of Corn Trypsin Inhibitor (CTI) or the carrier, and incubated at 37 °C for 10 minutes. Subsequently, blood samples were incubated with 0.066 mg_Fe_ mL^−1^ IONPs at 37 °C for 5 min, re-calcified with 20 mM CaCl_2_, stimulated either with 20 μL of a commercial solution of thromboplastin (Phosphoplastin RL, Enzyme Research Laboratory UK) or a similar volume of HBS, and placed in the sample holder inside the instrument.

### Clotting time measurements

500 μL of whole blood samples placed in plastic cuvettes were pre-treated with 50 μg mL^−1^ CTI or the carrier and incubated at 37 °C for 10 min. After that, the samples were added either with (i) 0.066 mg_Fe_ mL^−1^ of IONPs, (ii) a similar concentration of IONPs previously suspended in a 5% (w/v) solution of Bovine Serum Albumin (BSA, Sigma, UK) or (iii) the carrier. Immediately after, samples were re-calcified with 20 mM CaCl_2_ and kept at 37 °C. Clotting time was then measured visually by inverting the cuvettes and recording the time when the clot remained stuck at the bottom of the cuvette.

### Statistics

Blood from two to three donors were used in each experiment unless stated otherwise. *n* signifies the number of independently tested samples. Values are reported as mean ± standard error of the mean (SEM), except for IONPs morphological characterisation, which are mean ± standard deviation (SD). Statistical significance was assessed by one-way ANOVA for repeated measurements with a *post hoc* Tukey test using OriginLab Pro 8.5 software. A *P* < 0.05 value was considered statistically significant.

## Results and discussion

### Physico-chemical properties of the magnetic nanoparticles

**Fig. 2:**
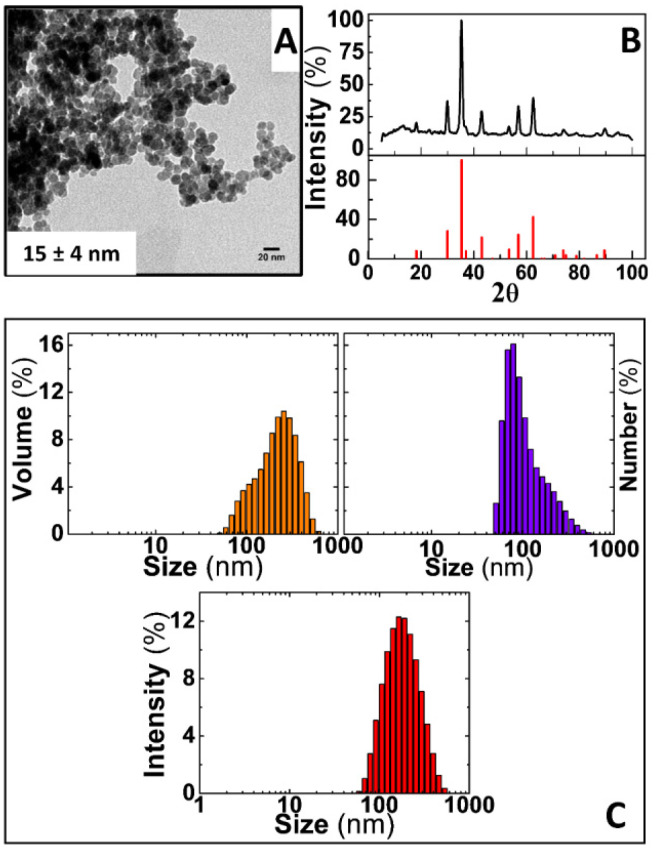
Physico-chemical characterisation of iron oxide nanoparticles. (A) Representative TEM image of sucrose coated IONPs. (B) XRD diffractogram of IONPs performed in dried powders, iron oxide COD: 96-900-9769 (C) DLS diagrams of IONPs dispersed in ddH_2_O and weighted by volume (orange columns), number (purple columns) and intensity (red columns). Scale bar in (A) = 20 nm.

Prior to assessing the capabilities of IONPs and AC magnetic susceptibility for tracking coagulation in freshly donated human whole blood, the physical and chemical properties of IONPs were assessed by TEM, XRD, DLS and Nanoparticle Tracking Analysis (NTA). These nanoparticles consist of in-house synthesised IONPs coated with sucrose using a co-precipitation method of iron salts under basic conditions, as described in the experimental section. Fig. 2A shows a representative TEM image of IONPs, unveiling an irregular rounded morphology as usually observed in nanoparticles produced by this synthesis route, and a core size distribution of 15 ± 4 nm. Crystal structure of magnetic cores was determined by XRD. As shown in Fig. 2B, a diffraction pattern with well-defined peaks were obtained from dried IONPs powders. This pattern can be indexed to that of magnetite (COD: 96-900-9769) although this same pattern can be indexed also to γ-Fe_2_O_3_ or Fe_3_O_4_. Since both phases account for the same unit cubic structure, such indexing is insufficient for a reliable determination of the main phase present in the IONPs cores. To this aim, a Rietveld analysis was performed. Assuming the crystal structure of magnetite,^25^ the Rietveld analysis provided a lattice parameter (*a*) for a cubic cell with a space group *Fm*3*m* equal to 8.380 Å, which is much closer to that associated to magnetite phase (8.396–8.40 Å) rather than that of maghemite (8.33–8.34 Å).^26^ Further to this, the Rietveld analysis shed a crystallite distribution size of 13.4 nm ± 5 nm for IONPs, which is in good agreement with that obtained by TEM. Thus, this analysis suggests that magnetite is the main constituent of the IONPs cores. DLS measurements of IONPs in ddH_2_O retrieved *z*-average values of 163.4 nm with a polydispersity (PDI) of 0.182, as well as 164.2, 78.82 and 255 nm of *D*_H_ weighted in intensity, number and volume, respectively, as shown in Fig. 2C.

Complementary analysis performed by NTA reasonably overlapped with the results obtained by DLS by providing a mean hydrodynamic size of 152.8 ± 48.2 nm (Fig. S1†). All these values are in agreement with nanoparticles previously obtained through similar methods of chemical synthesis.^21,27^

### IONPs magnetic behaviour is dominated by Brownian relaxation mechanism when dispersed in aqueous solutions

For monitoring blood coagulation dynamics using the principles of AC magnetic susceptibility, Brownian relaxation mechanism must be the predominant magnetic behaviour of the IONPs colloids. Upon blood clotting there will be a progressive increase in blood viscosity hindering IONPs mobility, and an enlarged hydrodynamic size *D*_H_ in IONPs, mediated by the IONPs getting trapped in the forming clot through platelet aggregation and formation of a fibrin mesh.^28^ These changes would modify Brownian relaxation time and be reflected in the AC magnetic susceptibility signal over time. Thus, measuring AC magnetic susceptibility in whole blood samples, labelled with an IONPs that predominantly feature Brownian relaxation, would provide the basis for a real-time measurement of clotting in whole blood.

**Fig. 3:**
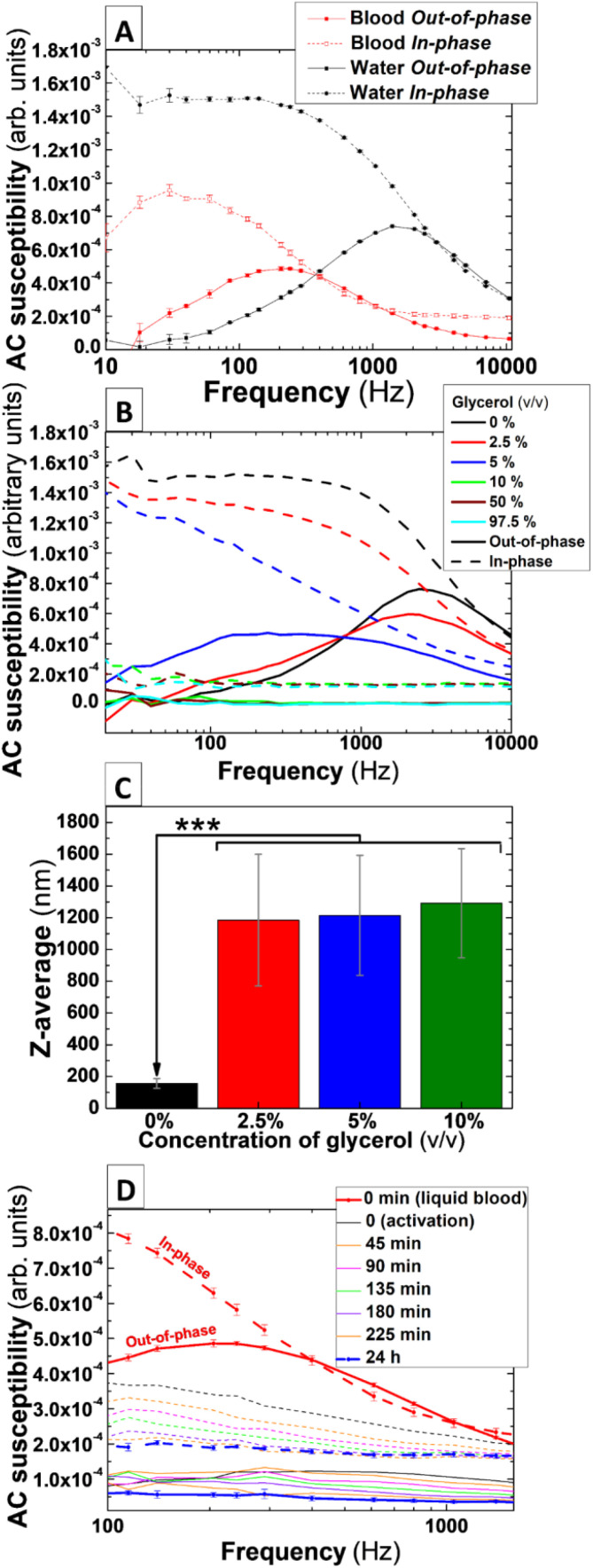
AC susceptibility traces varies according to an increase in nanoparticles aggregation and the viscosity of solution media and freshly donated human whole blood. (A) AC susceptibility measurements of 0.066 mg_Fe_ mL^−1^ IONPs suspended in water, freshly donated whole human blood and (B) mixed solutions of water and glycerol 2.5, 5, 10, 97.5% (v/v). (C) DLS measurements of 0.066 mg_Fe_ mL^−1^ IONPs dispersed in either water or mixed solutions of water and 2.5, 5 and 10% glycerol (v/v). (D) AC susceptibility measurements of 0.066 mg_Fe_ mL^−1^ IONPs suspended in freshly donated whole human blood recalcified with 20 mM CaCl_2_ and activated by addition of 125 μM ADP. *n* = 3 in (A), (C), and (D) at *t* = 0 (liquid blood) and *t* = 24 h. ***P < 0.001.

To initially determine the relaxation mechanism driving the magnetic response of the IONPs in liquids, AC magnetic susceptibility measurements of IONPs suspended in ddH_2_O were performed in spectral frequency mode (see Experimental section). As shown in Fig. 3A (black lines), AC susceptibility measurements of IONPs suspended in ddH_2_O displayed a well-defined peak with a maximum at 2 kHz in the out-of-phase component. Such a signature in susceptibility could be associated with Brownian relaxation, according to results from IONPs with similar features previously published elsewhere.^20,29^ Remarkably, the intersection between the in-phase and out-of-phase components of the AC magnetic susceptibility occurs in the proximity of the maximum of the Brownian peak. This crossover suggests that most of the particles along the size distribution of the IONPs behaves according to a Brownian relaxation pattern.^30^ To further assess whether the observed peak is mainly due to Brownian relaxation, AC magnetic susceptibility measurements of IONPs were performed in aqueous solution with increasing concentrations of glycerol to progressively induce the immobilisation of IONPs and therefore reduce the Brownian relaxation time *τ*_B_. Fig. 3B shows AC susceptibility measurements of IONPs suspended in mixed aqueous solutions ranging from 0 to 97.5% glycerol (v/v). At first glance, the Brownian peak of the nanoparticles measured only in ddH_2_O (0% glycerol) appears slightly shifted to higher frequencies in comparison to its counterpart in Fig. 3A. This could be due to slight variations in the hydrodynamic size of IONPs between experiments. Also, a glycerol fraction-dependence shift of the peak towards lower frequencies is observed in the susceptibility curves, with the peak appearing to be totally suppressed in glycerol concentrations over 10% (v/v). This behaviour is compatible with a predominance of a Brownian relaxation mechanism driving the magnetic dynamics of the studied IONPs colloids, as Brownian relaxation time is directly proportional to the viscosity of the liquid media in which nanoparticles are suspended. Interestingly, the peak associated with Brownian relaxation also showed a widening from the smallest fraction of glycerol utilised in the measurements (Fig. 3B-red and blue solid line). This contrasts with results in previous studies where IONPs of similar size and composition (albeit coated with citric acid), uniquely exhibited a displacement of the Brownian peak towards lower frequencies with no widening at increased glycerol fractions in the carrier solution.^20^ However, note that previous DLS measurements from those citrated nanoparticles showed no changes in the *D*_H_ and PDI in solutions containing glycerol, suggesting that the use of sucrose as a coating agent might make IONPs from the present work more susceptible to variations in *D*_H_ and PDI when dispersed in glycerol-containing media.

To assess whether glycerol triggers aggregation of the IONPs, DLS measurement of IONPs in aqueous solutions containing glycerol fractions were performed. Note that the equipment can measure *D*_H_ in aqueous solution containing a maximum of 25% glycerol (v/v) and therefore measurements were uniquely performed for those suspensions with a glycerol fraction below this upper limit. As shown in Fig. 3, glycerol triggers a significant rise in *D*_H_ and PDI in mixed solvent solutions including at the lowest glycerol fraction of 2.5% (v/v) (Fig. 3C, and Table 1). This significant rise in both colloidal parameters *versus* the control (*P* < 0.001) explains the progressive widening observed in the out-of-phase peak in solutions with increased glycerol content.

**Table 1:** Hydrodynamic size of IONPs measured in aqueous solution containing increasing fractions of glycerol

Glycerol fraction (% v/v)	Hydrodynamic size/*z*-average (nm)	PDI	Standard error of the mean (SEM)
0	156.3	0.117	30.9
2.5	1185	0.367	414.5
5	1292	0.213	344.3
10	1215	0.29	377.8

### Frequency spectral AC magnetic susceptibility measurements reveal variations compatible with blood coagulation dynamics

IONPs generally undergo increased *D*_H_ values when suspended into whole blood. Due to the non-selective absorption of circulating biomolecules onto the nanoparticles surface, a protein corona is formed immediately after these are exposed to blood plasma.^31^ This, together with the high ionic strength of blood plasma, induce the formation of larger IONP clusters when dispersed in blood. This might result in the Brownian peak observed when IONPs are suspended in ddH_2_O (Fig. 3A) being displaced to frequencies below 1 Hz, which is beyond the detection limit of the AC magnetic susceptometer. To rule out this possibility, AC magnetic susceptibility measurements in frequency spectral mode were performed in non-activated (*i.e.* citrated) freshly-donated human whole blood containing IONPs. As shown in Fig. 3A (solid red line), the maximum of the peak in out-of-phase susceptibility appears shifted towards lower frequencies (∼200 Hz) when compared to the measurements of IONPs in ddH_2_O suspension (∼1.5 kHz). Further to this, the intensity of the signal in the in-phase and out-of-phase components is reduced by approximately one third when IONPs are suspended in blood, which suggests the occurrence of IONPs aggregation.^20^ Remarkably, the shift of the peak and decrease in AC susceptibility signal, induced by both the formation of a protein corona on the nanoparticles surface and the ionic strength of blood, did not relocate the peak beyond the detection range of the instrument. Therefore, AC magnetic susceptibility signal of these IONPs could feasibly be used to monitor blood coagulation.

To initially assess whether AC susceptometry and IONPs could monitor blood clotting dynamics, we performed frequency spectral susceptibility experiments in freshly donated whole blood after triggering thrombus formation using the platelet agonist ADP. Fig. 3D shows AC magnetic susceptibility measurement of IONPs-labelled whole blood before activation (Fig. 3D-red lines), consecutive measurements every 45 min for 3 h after ADP stimulation in whole blood from a single donor, and a final measurement 24 hours after activation. Activation of blood coagulation produces a prominent fall in both the in- and out-of-phase AC magnetic susceptibility as shown in the initial measurements (Fig. 3D-black lines). This abrupt flattening after activation of blood clotting suggests an initial strong immobilisation and aggregation of the IONPs inside the developing clot in a manner analogue to the behaviour observed in measurements using mixed solutions of ddH_2_O and glycerol (Fig. 3B). This trend is observed in repeated measurements performed each 45 minutes until no significant signal is detected in the out-of-phase component of the susceptibility of IONPs in fully compacted clots after 24 h (Fig. 3D-blue lines), pointing to a significant aggregation and immobilisation of IONPs inside the blood clot structure. These results demonstrate the link between the AC magnetic susceptibility signal from IONPs and the coagulation dynamics of freshly donated human whole blood.

### Single frequency AC magnetic susceptibility can monitor *ex vivo* the effects of indirect anticoagulants in coagulation dynamics of human whole blood

Unfractionated heparin is an anticoagulant drug containing a pool of anionic, sulphated glycosaminoglycan polymers. Heparin binds reversibly to antithrombin III, which can then bind to and inhibit thrombin and factor Xa in the coagulation cascade. Through this mechanism of action, heparin prevents thrombin formation and activity – inhibiting fibrin formation. This anti-coagulant drug is used to treat conditions such as VTE^32^ and atrial fibrillation.^33^ People treated with heparin require close monitoring^34^ due to increased risk of bleeding^35^ or osteoporosis if used long term in patients.^36^ To assess whether heparin-induced inhibition of blood coagulation could be observed in AC magnetic susceptibility, measurements were performed in blood samples labelled with IONPs and pre-treated with 0.5, 15 and 30 U mL^−1^ of unfractionated heparin or its carrier (control). Note that AC magnetic susceptibility measurements in frequency spectral mode for the previous section showed significant variations in the out-of-phase component over time at 400 Hz (Fig. 3D). To perform faster AC magnetic susceptibility measurements while keeping enough resolution in the out-of-phase related to variations in blood coagulation, measurements were performed in a single frequency mode at 400 Hz (see Experimental section).

**Fig. 4:**
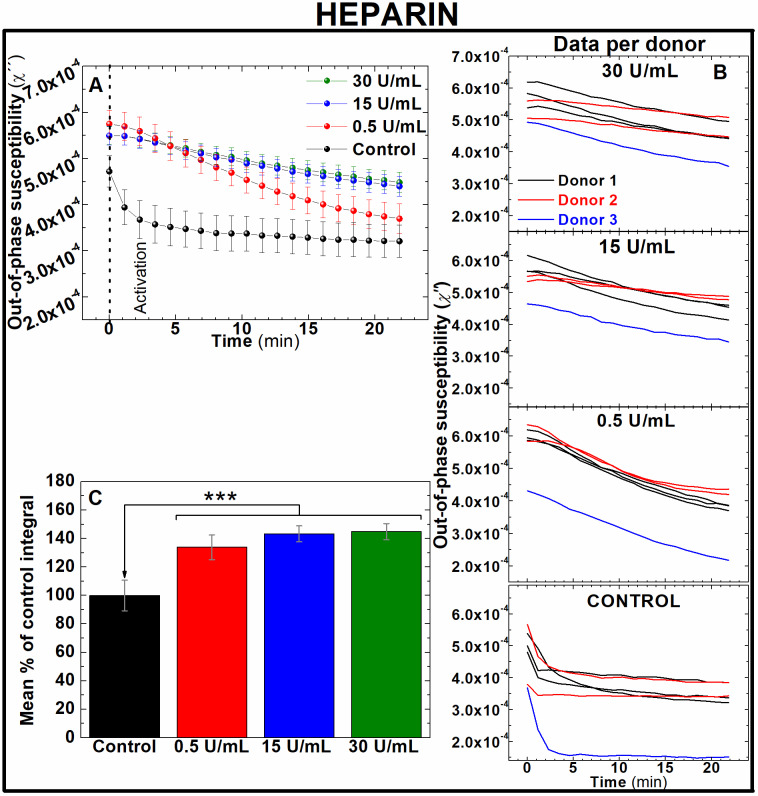
Out-of-phase component from AC magnetic susceptibility recorded at 400 Hz enables to identify the effect of different doses of heparin in freshly donated human whole blood. (A) Average traces and (B) traces per human donor of out-of-phase component from AC magnetic susceptibility measurements of 0.066 mg_Fe_ mL^−1^ IONPs suspended in freshly donated whole human blood, pre-treated with 0.5, 15 and 30 U mL^−1^ heparin, recalcified with 20 mM CaCl_2_ and activated with 125 μM ADP (C) area enclosed below AC susceptibility traces measured in (B). *n* = 6 ***P < 0.001. Error bars: SEM.

Fig. 4A and B show, respectively, the average and per donor measurements of the out-of-phase component of susceptibility in heparin pre-treated human whole blood. All the traces depict a sustained fall over time. While untreated samples depict an exponential decrease in susceptibility just after clotting activation, this rate of decrease slows down in samples treated with higher concentrations of heparin. This agrees with the anticoagulant effects of heparin to slow blood clotting. By indirectly inhibiting thrombin and factor Xa in a dose-dependent manner, clot formation is reduced and therefore also aggregation and immobilisation of IONPs inside the thrombus. To quantify differences between the effectiveness of pre-treatments, areas enclosed under the curve of the susceptibility measurement of the out-of-phase component over time were calculated for each heparin dose. As shown in Fig. 4C, significant differences were observed between all the heparin-treated blood samples and untreated control samples (133.9 ± 8.7, 143.27 ± 5.6 and 144.86 ± 5.66% of control for 0.5, 15 and 30 U mL^−1^ of heparin respectively, *n* = 6, *P* < 0.001). Remarkably, the lowest dose utilised in these experiments is below the recommended intravenous loading doses. According to the National Institute for Health and Care Excellence (NICE) in the UK, 1–2 units of heparin per mL of blood (U mL^−1^) are recommended as an initial loading dose in clinical scenarios such as VTE and thromboprophylaxis. This demonstrates that AC magnetic susceptibility can detect the effect of heparin in blood coagulation even at subtherapeutic doses.

### AC magnetic susceptibility can monitor the effect of direct anticoagulants in whole human blood

**Fig. 5:**
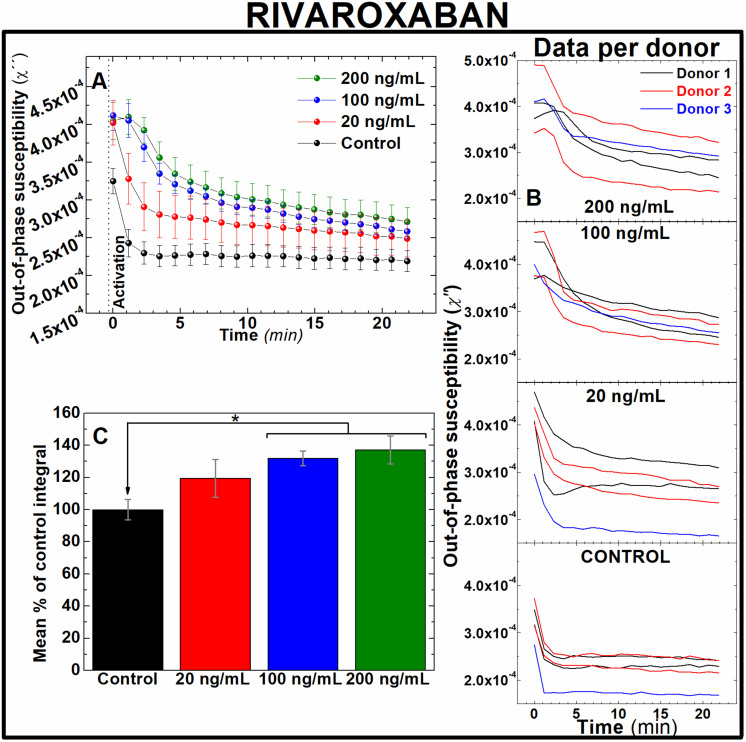
Out-of-phase component from AC magnetic susceptibility recorded at 400 Hz enables to identify the effect of different doses of rivaroxaban in freshly donated human whole blood. (A) Average traces and (B) traces per human donor of out-of-phase component from AC magnetic susceptibility measurements of 0.066 mg_Fe_ mL^−1^ IONPs suspended in freshly donated whole human blood, pre-treated with 20, 100 and 200 ng mL^−1^ rivaroxaban, recalcified with 20 mM CaCl_2_ and activated with 125 μM ADP (C) area enclosed below AC susceptibility traces measured in (B). *n* = 5, *P < 0.05, error bars: SEM.

**Fig. 6:**
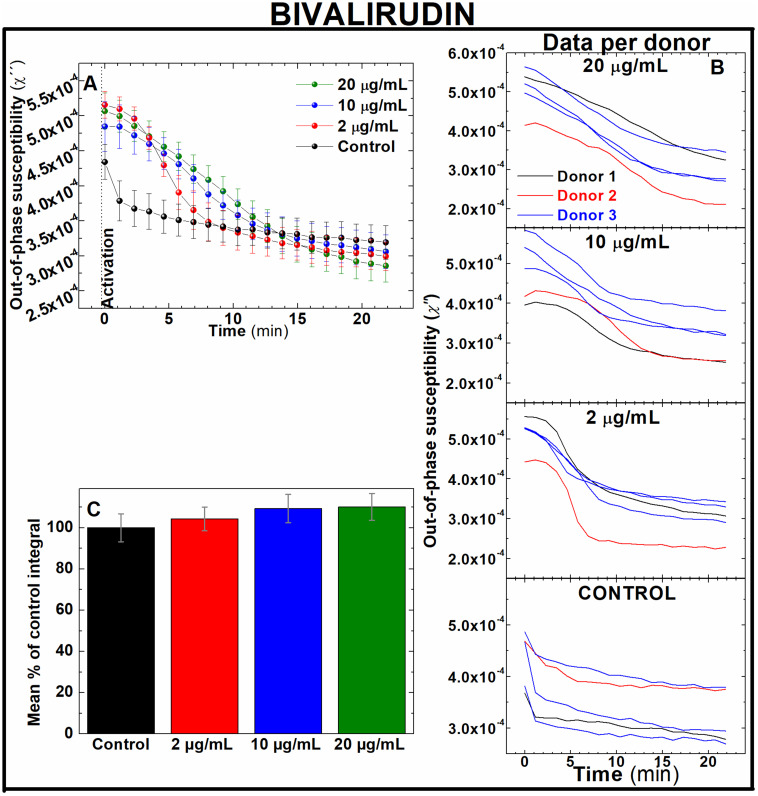
Out-of-phase component from AC magnetic susceptibility recorded at 400 Hz enables to identify the effect of different doses of bivalirudin in freshly donated human whole blood. (A) Average traces and (B) traces per human donor of out-of-phase component from AC magnetic susceptibility measurements of 0.066 mg_Fe_ mL^−1^ IONPs suspended in freshly donated whole human blood, pre-treated with 2, 10 and 20 μg mL^−1^ bivalirudin, recalcified with 20 mM CaCl_2_ and activated with 125 μM ADP (C) area enclosed below AC susceptibility traces measured in (B). *n* = 5, error bars: SEM.

Rivaroxaban and bivalirudin are clinically used anticoagulant drugs that in contrast to unfractionated heparin, exert a direct, highly selective inhibition of a single coagulation factor in the coagulation cascade. While rivaroxaban selectively inhibits the active form of factor X (factor Xa),^37^ bivalirudin is a direct thrombin inhibitor.^38^ To assess whether AC magnetic susceptibility can track the effect of these drugs in blood coagulation, measurements of the out-of-component signal of AC magnetic susceptibility were performed *ex vivo* in freshly donated human whole blood samples. Following guidelines of clinical doses for rivaroxaban^39^ and bivalirudin,^40^ IONPs-labelled blood samples were pre-treated with either 20, 100 and 200 ng mL^−1^ of rivaroxaban (Fig. 5) or 2, 10 and 20 μg mL^−1^ of bivalirudin (Fig. 6). These doses correspond to effective plasma concentrations found in patients taking these drugs.^39^ As expected from previous experiments, the out-of-phase component of AC magnetic susceptibility described a significant decrease in control samples after activation of blood coagulation over time (Fig. 5A and 6A, black line). In a similar way, the evolution of the out-of-phase component over time for each anticoagulant drug varied according to the dose of drug utilised in the blood pre-treatment. Incremented doses of rivaroxaban (Fig. 5A) or bivalirudin (Fig. 6A) reduced the rate of decrease of the out-of-phase susceptibility in a dose-dependent manner, although this effect is observed to a lesser extent when compared with samples pre-treated with heparin (Fig. 4). The higher selectivity of rivaroxaban and bivalirudin over heparin to inhibit coagulation factors, as well as the short time window where bivalirudin is still effective slowing coagulation (typically less than 25 minutes),^41^ explains the diminished effect of these drugs on blood coagulation. The anticoagulant effect can be also observed in the area under the out-of-phase component of AC susceptibility curves. As shown in Fig. 5C, the effect on coagulation from clinical doses of rivaroxaban (100 and 200 ng mL^−1^) are significantly different from control (131.8 ± 4.5 and 137.2 ± 8.8% of control respectively, *n* = 5, *P* < 0.05). In addition, differences in shape and intensity in out-of-phase susceptibility traces over time still enables to unveil the presence of the anticoagulant in blood at doses of 20 ng mL^−1^ when compared to control (Fig. 5A), even if the areas between these measurements are not significantly different (Fig. 5C). This is also the case for blood pre-treated with bivalirudin. Here, no significant differences are found between the areas of traces in pre-treated blood with control (Fig. 6C), although traces evidently vary in shape and evolution over time from control.

Direct oral anticoagulants (DOAC) like rivaroxaban were designed to reduce the need of frequent coagulation monitoring in patients^42^ suffering from conditions like atrial fibrillation,^43^ coronary heart disease^44^ and VTE,^45^ as well as those in need of thromboprophylaxis.^46^ However, monitoring of blood coagulation is still required for a number of demographic groups taking these drugs. These include kids, pregnant women and elderly people, as well as patients with extreme body weight, suffering from significant bleeding or active thromboembolism, undergoing invasive surgical procedures, or suspected from non-compliance or overdose whilst under DOAC prescription.^47–51^ In contrast bivalirudin, used in percutaneous coronary intervention procedures^52^ and patients with heparin-induced thrombocytopenia at high risk of bleeding,^53^ requires of frequent coagulation monitoring.^54^ Therefore, fast, sensitive techniques able to accurately assess efficacy of these drugs on coagulation are still required in these frequently occurred clinical scenarios. Our results suggest that this new technology could be utilised in clinical scenarios where rapid testing of new generation of anticoagulant drugs is needed. Also, this data demonstrate the sensitivity of this approach to detect the effect of DOAC at therapeutic doses in human whole blood (Fig. 5), in contrast to the incapability of routine tests PT/PTT to reliably monitor the effect of DOACs in blood coagulation.^8^

### AC magnetic susceptibility beyond anticoagulation therapy: antiplatelet drugs

**Fig. 7:**
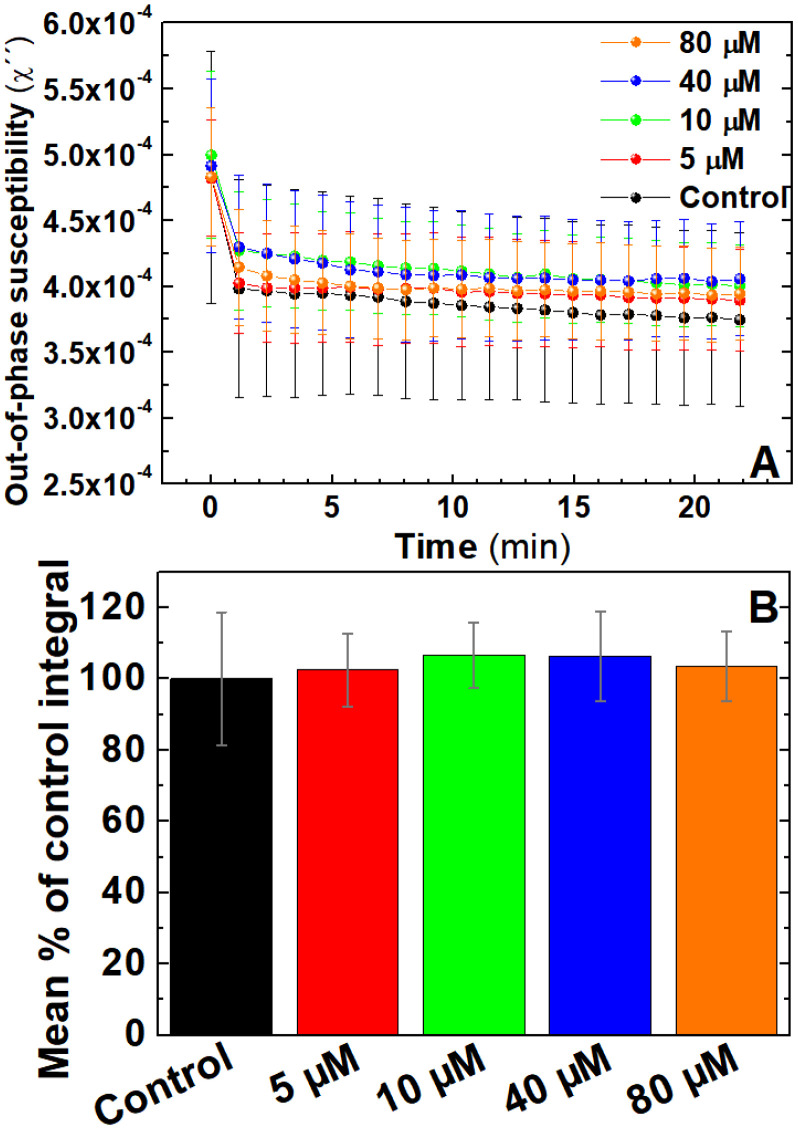
Out-of-phase component from AC magnetic susceptibility recorded at 400 Hz shows no significant differences in samples pre-treated with anti-platelet drug MRS2179. (A) Average traces of out-of-phase component from AC magnetic susceptibility measurements of 0.066 mg_Fe_ mL^−1^ IONPs suspended in freshly donated whole human blood, pre-treated with 5, 10, 40 and 80 μM MRS2179 or the carrier, recalcified with 20 mM CaCl_2_ and activated with 125 μM ADP. (B) Area enclosed below AC susceptibility traces measured in (B). *n* = 5. Error bars: SEM.

Antiplatelets are antithrombotic drugs that prevent clot formation by inhibiting platelet function.^55,56^ In contrast to anticoagulants generally used for conditions involving stoppage of the normal flow of fluids, antiplatelets drugs are utilised in conditions where endothelial dysfunction promotes unwanted platelet activation such as coronary artery disease.^57^ To assess the ability of AC magnetic susceptibility for monitoring the effect of antiplatelets drugs in blood coagulation, AC magnetic susceptibility was performed in freshly-donated whole blood labelled with IONPs and pre-treated with the antiplatelet drug MRS2179^58^ using doses of 5, 10, 40 and 80 μM or the drug carrier. As shown in Fig. 7A, no clear differences were observed between the out-of-phase traces from different doses. This is also portrayed in the analysed areas of the traces (Fig. 7B) where no significant differences were observed between groups. In addition to this, further pilot measurements using high doses of MRS2179 in combination with clinically used antiplatelet drugs such aspirin and ticagrelor showed insignificant differences (Fig. S2†). All this data suggests that the current monitoring capabilities of AC susceptometry used in this study are selective for changes in the blood sample caused by blood coagulation and not platelet activation.^56^ However, detection of platelet activation may be possible through these techniques by improving the instrumentation, platelet agonist and optimising the magnetic nanoparticles used. The inability of activating blood clotting while the sample is inside the instrument impedes our ability to monitor changes during the rapid initial timeframe in which thrombus formation is driven by platelet activation and aggregation before the onset of coagulation. Additionally, our inability to pick up platelet activation in the AC magnetic susceptometer may be impacted by our choice of a weak platelet agonist such as ADP, whose activity may be affected by the ectonucleotidases found on red blood cells. Such choice would allow thrombin formed from the activation of the coagulation cascade to dominate and ensure that platelet activation and fibrin formation occur simultaneously, leading to the role of platelets being masked in these studies. The use of stronger, platelet-specific agonists such as collagen-related peptide or thrombin receptor activating peptides may provide a more effective method to assess if platelet activation can also be detected by this magnetic coagulometer. In addition, this is further impacted by the volumetric proportion of red blood cells which make up a much greater proportion of the blood volume compared to platelets, thus potentially masking the role of platelet aggregation in blood clot formation. Further developments such as the functionalisation of the IONPs with PAC-1 antibody, as in our previous works,^21^ could help to specifically relate the susceptibility signal to activated platelets. This would eventually allow to effectively monitor primary coagulation, related to platelets, in a complex media of whole blood.

### Magnetic coagulometry can monitor coagulation triggered by either intrinsic or extrinsic coagulation pathways

Two main pathways are involved in coagulation: the intrinsic and extrinsic pathways. These pathways converge at a final common pathway to trigger thrombin activation and fibrin formation that allows the formation of a stable blood clot. The activation of the extrinsic pathway requires exposure of the bloodstream to tissue factor, present in the exposed subendothelial matrix following vascular damage. In contrast, the activation of the intrinsic pathway requires activation of clotting factors already present in blood by negatively charged surfaces found in thrombotic and inflammatory environments. One of the factors involved in the intrinsic pathway, Factor XII, has previously been shown to be activated by negatively charged nanoparticles^59^ and trigger an enzymatic cascade that activates the intrinsic pathway. Therefore experiments were performed to assess if the magnetic nanoparticles used in this study could be influencing the rate of blood coagulation through artificially activating the intrinsic pathway.

**Fig. 8:**
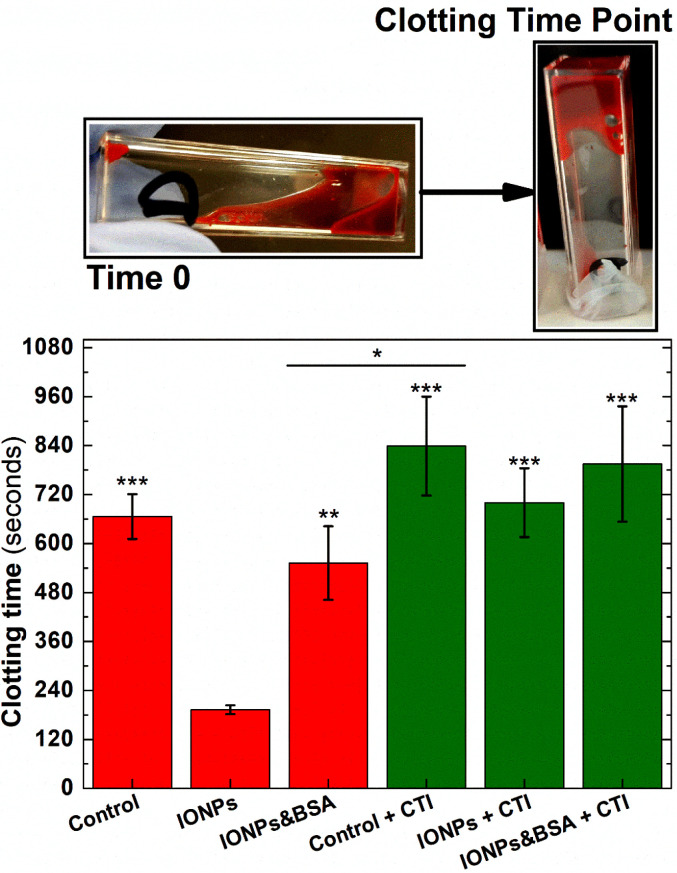
Clotting times in freshly donated human whole blood labelled demonstrate that IONPs activates the intrinsic pathway of coagulation. Whole Blood samples pretreated with 50 μg mL^−1^ CTI (green bars) or the carrier (red bars). Whole blood samples incubated with 0.066 mg_Fe_ mL^−1^ IONPs (IONPs bars), similar concentration of IONPs pre-treated with BSA solution (BSA bars), or the carrier (control bars). Images above the graph represent the initial and final point of clotting time experiments. *n* = 5, ***P < 0.001 *vs.* IONPs group, **P < 0.01 *vs.* IONPs group, *P < 0.05 between groups. Error bars = SEM.

Clotting time experiments were performed in whole blood samples pretreated with CTI, a selective inhibitor of the activated form of Factor XII.^60^ As shown in Fig. 8, clotting time after blood recalcification was significantly shortened when whole blood was pre-incubated with IONPs when compared with the control (192 ± 11 and 666 ± 55 s, respectively, *n* = 5, *P* < 0.001). However, this effect disappeared when the intrinsic pathway was inhibited by pre-treating blood with CTI. As observed in Fig. 8, measurements of clotting time in whole blood pre-treated with CTI and incubated with IONPs (IONP + CTI) remains insignificantly different from both untreated whole blood (Control) and from whole blood pre-treated only with CTI (Control + CTI). Albumin has previously been used to produce a passivating layer on biomaterials to help ensure they are haemocompatible by preventing activation of the foreign body response.^61^ As shown in Fig. 8, pre-treatment of the IONPs with BSA (IONPs & BSA) prevents the enhanced clotting time seen in untreated IONPs. The clotting times of BSA-treated particles were not significantly enhanced by pretreatment with CTI (IONPs&BSA + CTI). These results indicate that the IONPs used in the measurement enhanced the coagulation observed through activation of the intrinsic pathway of coagulation.

**Fig. 9:**
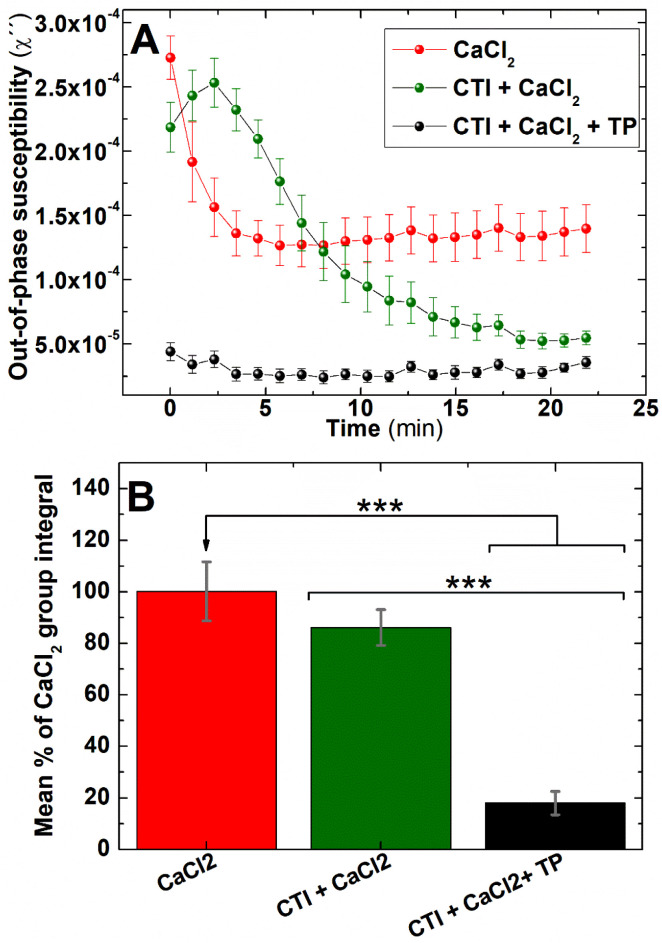
Out-of-phase component from AC magnetic susceptibility recorded at 400 Hz can identify the contribution of the extrinsic pathway in coagulation in freshly donated human whole blood. (A) Whole blood samples pre-treated with 50 μg mL^−1^ CTI (green and black traces) or the carrier (red traces) and re-calcified with 20 mM CaCl_2_ (all traces) and stimulated with 20 μL of thromboplastin (black traces) or saline solution (red and green traces). (B) Area enclosed below AC susceptibility traces measured in (A). *n* = 6, ***P < 0.001, error bars = SEM.

To determine if the IONPs are also able to also detect coagulation elicited by activation of the extrinsic pathway of coagulation, AC magnetic susceptibility measurements were performed in IONPs-labelled freshly donated human whole blood (i) without further treatment, (ii) pre-treated with CTI, or (iii) pre-treated with CTI and activated *via* extrinsic pathway by adding a reagent containing tissue factor. Single frequency out-of-phase susceptibility measurements showed an abrupt fall after recalcification when samples were pre-treated only with IONPs (Fig. 9A-red line). However, this fall in susceptibility was strongly softened when samples were pre-treated with the inhibitor of the intrinsic coagulation pathway CTI (Fig. 9A-green line). This is consistent with the IONPs-mediated activation of the intrinsic pathway observed in clotting time experiments (Fig. 8). Although IONPs-labelled blood samples eventually clot without the addition of an activator in both experiments, this coagulation dynamics is significantly delayed when the IONP-mediated activation of the intrinsic pathway is inhibited by pre-treating of blood samples with CTI. Also, IONPs-mediated activation of intrinsic coagulation pathway was observed in frequency spectral mode (Fig. S3†). To assess if activation of the extrinsic pathway could still be observed in these samples, we monitored the effect of addition of thromboplastin (an exogenous activator of the extrinsic pathway) into the sample. The out-of-phase susceptibility of IONPs-labelled whole blood samples pre-treated with CTI and activated with thromboplastin showed an extremely rapid drop (Fig. 9A-black trace), indicating that co-activation of the extrinsic pathway alongside the activation of the intrinsic pathway by the IONPs allows for a more rapid coagulation. This data strongly suggests both that magnetic coagulometry can monitor blood coagulation driven by activation of the extrinsic pathways of coagulation, and that IONPs-mediated activation of blood is not required for the system to monitor coagulation. Also, this experimental data reinforces the hypothesis that it is overall blood coagulation, and not solely the IONPs-mediated activation of the intrinsic pathway, that can be detected through these out-of-phase susceptibility measurements. They also suggest that whilst the data support the overall capability of magnetic coagulometry to measure coagulation, further work will be required to produce a passivated IONP that can act solely as a sensor of coagulation without directly influencing it as seen in these studies.

### State of the art and further developments

PT and aPTT provide invaluable information in a research and clinical setting about the potential activity of the extrinsic and intrinsic pathways of coagulation respectively. PT/aPTT are performed by mixing platelet-poor plasma (blood samples deprived of significant blood components like platelets and red blood cells) with a commercial activating reagent such as thromboplastin or kaolin. While PT/aPTT are useful for testing secondary haemostasis, they utilise isolated plasma and provide limited information beyond the basic biochemical properties of these cascades.^62^ Similarly, platelet aggregometry measures platelet activation through changes in light transmission through purified samples with a certain degree of transparency, such as washed platelets or platelet-rich plasma. However this technique provides no information on blood coagulation and overall blood clot structure in whole blood as can be achieved in our nanotechnological approach. Given the heterogeneous character of clot structure,^63^ as well as the intricate role of blood components modulating coagulation^4^ especially in cases of abnormal thrombogenesis, further developments of a nanotechnological approach could provide a powerful tool to understand the complex interplay of coagulation mechanisms in haemostatic disorders and significantly improve their diagnosis and treatment. For instance, magnetic nanoparticles could be passivated to prevent the activation of the intrinsic pathway and functionalised with peptides and antibodies to target discrete cell and biomolecular components in forming clots. This would focus the magnetic probes on the behaviour of specific components to ascertain their contribution to clot formation. Here we demonstrated that IONPs can be passivated by pre-treating them with BSA (Fig. 8 – IONPs & BSA). Through the absorption of BSA on nanoparticle surface, clotting times of IONPs-labelled whole blood became non significantly different with control samples. This provides a foundation to conceive more complex nanoparticles selectively targeted to blood components. For instance, magnetic nanoparticles could be initially targeted to fibrinogen and become integrated in the fibrin mesh upon coagulation, therefore indicating any potential abnormality in the compaction pattern of the mesh through the out-of-phase signal from IONPs. Our results also suggest that magnetic coagulometry is able to track changes in the nanomechanical properties of blood clots in real-time. Due to the rudimentary macroscopic principle of action of thromboelastographic techniques to measure coagulation in whole blood (see Introduction section), all these principles could not be developed using these instruments. In addition, lower acquisition times in the instrument, and enhanced resolution of magnetic coagulometry would enable tracking both primary and secondary haemostasis. This would provide researchers with an experimental platform with the potential to selectively monitor coagulation in patients in a more detailed manner than the currently available testing methods.

## Conclusions

In this work, we have proposed and demonstrated a new magnetic coagulometry method to measure blood coagulation *ex vivo* in whole human blood. This new technique is based on the combination of AC magnetic susceptometry and purpose-designed IONPs that have optimal magnetic properties and enhanced Brownian response. By labelling freshly donated human blood with these specific nanoparticles, it is possible to monitor the effects on coagulation from three different commercial antithrombotic drugs by measuring the out-of-phase component of AC magnetic susceptibility. Our study potentially opens new horizons in whole blood coagulation testing and provide new grounds to overcome limitations of currently utilised methods for testing blood coagulation *ex vivo*.

## Author contributions

AH, NT, DO and DC secured funding for this research. ASO synthesised and characterised the iron oxide nanoparticles under the supervision of DO. DC performed the experiments. DC wrote the first draft of this manuscript, which included contributions and amendments from all the authors. All authors have given approval to the final version of the manuscript. Authors have no conflict of interests to disclose.

## Conflicts of interest

There are no conflicts to declare.

## Supplementary Material

NR-016-D3NR02593D-s001
