# Supplementary material for: Magnetic coagulometry: towards a new nanotechnological tool for ex vivo monitoring coagulation in human whole blood
Source: Nanoscale. 2023 Dec 21;16(7):3534–48. doi: 10.1039/d3nr02593d (PMC10868660; doi:10.1039/d3nr02593d)
Supplement: NR-016-D3NR02593D-s001 [file NR-016-D3NR02593D-s001.pdf]

Electronic Supplementary Material (ESI) for Nanoscale.
This journal is © The Royal Society of Chemistry 2023

Supporting Information

Magnetic Coagulometry: Towards a New Nanotechnological Tool for *ex vivo* Monitoring Coagulation in Human Whole Blood

Antonio Santana-Otero[a], Alan Harper[b], Neil Telling[c], Daniel Ortega[a,d,e], David Cabrera[c]

[a.] *Condensed Matter Physics Department, Faculty of Sciences, University of Cádiz. Campus Universitario Rio San Pedro s/n. 11510 Puerto Real (Cádiz) Spain*
[b.] *School of Medicine, Keele University, Newcastle-under-Lyme, Staffordshire. ST5 5BG, UK.*
[c.] *School of Pharmacy and Bioengineering, Keele University, Guy Hilton Research Centre, Thronburrow Drive, Hartshill, Stoke on Trent, ST47QB, UK*
[d.] *iMdea Nanociencia, Campus Universitario de Cantobalnco. C/Faraday, 9, 28049, Madrid, Spain.*
[e.] *Institute of Research and Innovation in Biomedical Sciences of Cádiz (INiBICA), University of Cádiz, 11002, Cádiz, Spain*

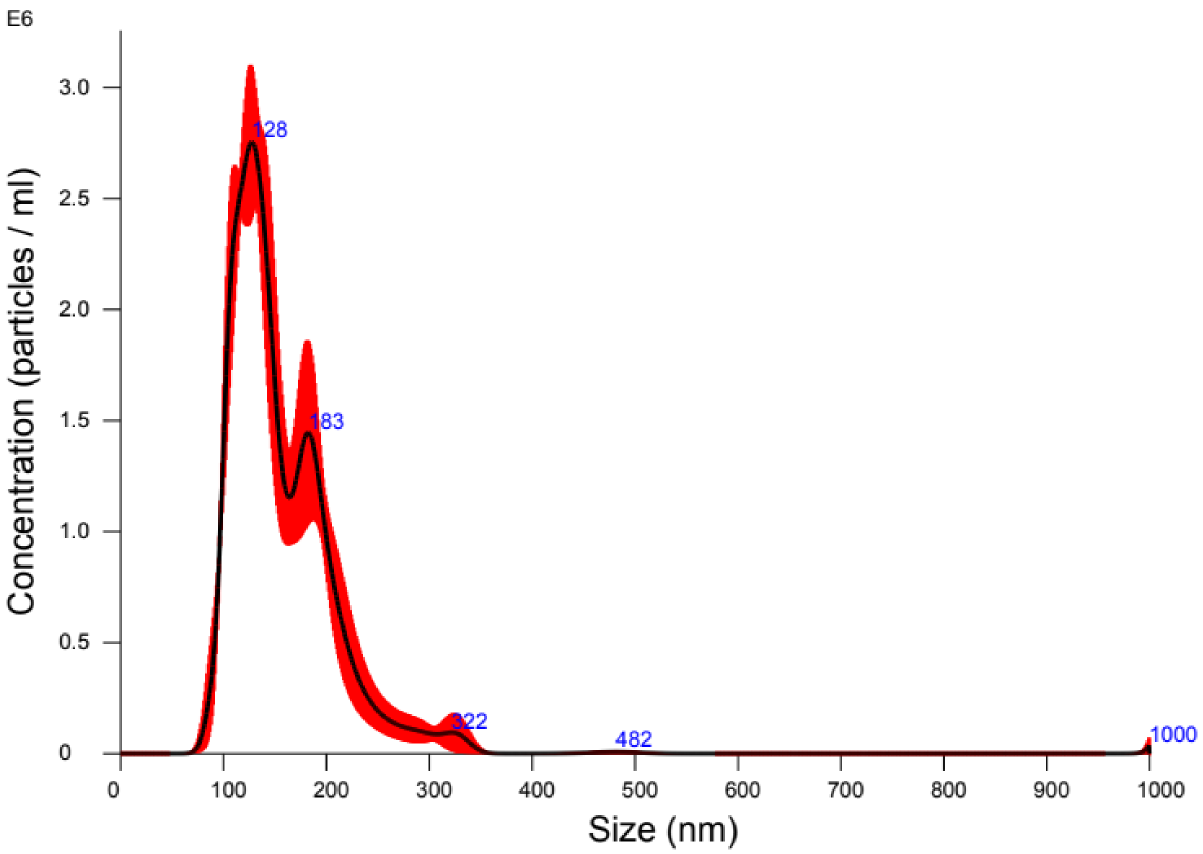

Figure S1. Nanoparticle Tracking Analysis diagram of IONPs.

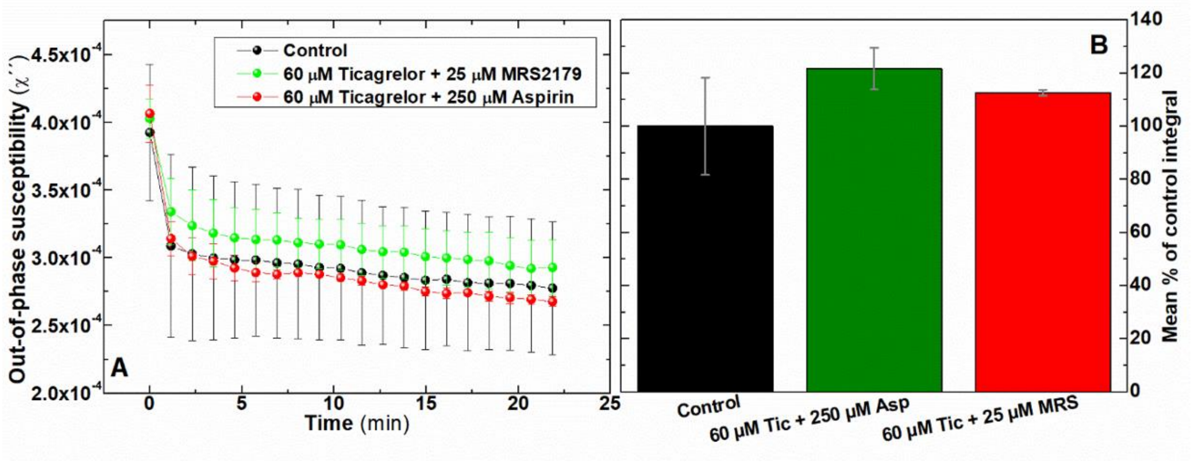

Figure S2. Preliminary out-of-phase component from AC magnetic susceptibility recorded at 400 Hz show no significant difference in samples pre-treated with combined anti-platelet drugs. (A) Average traces of out-of-phase component from AC magnetic susceptibility measurements of 0.066 mg_{Fe}/mL IONPs dissolved in freshly donated whole human blood, pre-treated with 60 µM Ticagrelor + 25 µM MRS2179, 60 µM Ticagrelor + 250 µM Aspirin or the carrier, recalcified with 20 mM $CaCl_2$ and activated with 125 µM ADP. (B) Area enclosed below AC susceptibility traces measured in (B). n = 3, 1 donor. Error bars: SEM

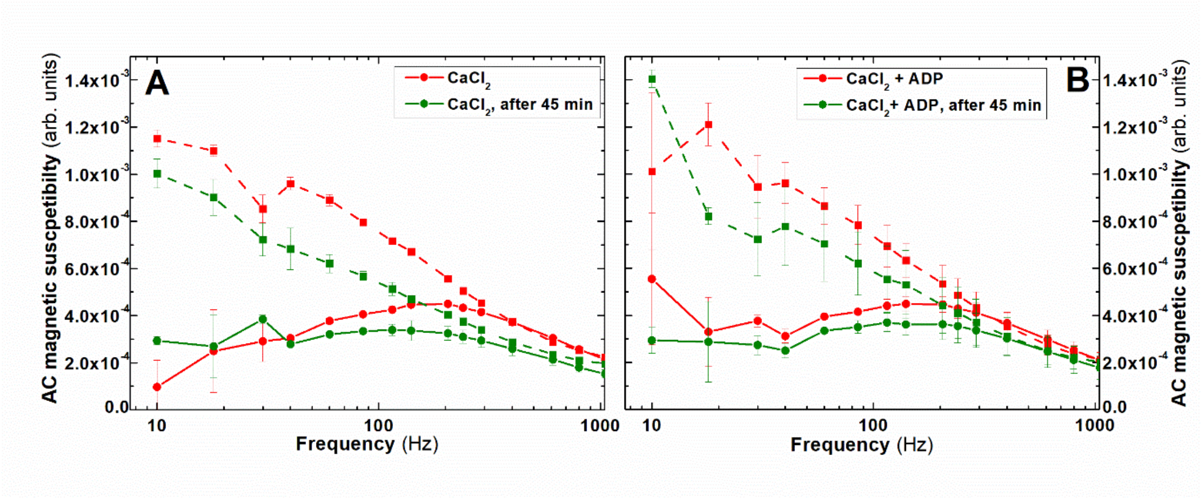

Figure S3. Frequency spectral mode AC susceptibility measurements of 0.066 mgFe/mL IONPs dissolved freshly donated whole human blood recalcified with 20 mM $CaCl_2$ and treated with (A) saline solution or (B) 125 µM ADP, recorded at time 0 and after 45 minutes. n = 2
